# Food Anaphylaxis: Eight Food Allergens Without Mandatory Labelling Highlighted by the French Allergy‐Vigilance Network

**DOI:** 10.1111/cea.70130

**Published:** 2025-08-20

**Authors:** Dominique Sabouraud‐Leclerc, Delphine Mariotte, Elena Bradatan, Amandine Divaret‐Chauveau, Carine Metz‐Favre, Pascale Beaumont, Pascale Dumond, Julien Serrier, Yasemin Karaca‐Altintas, Sélina Tscheiller, Guillaume Pouessel, Xavier Van der Brempt

**Affiliations:** ^1^ Department of Paediatrics AMH, CHU Reims Reims France; ^2^ Allergy‐Vigilance Network Nancy France; ^3^ Immunology and Histocompatibility Laboratory CHU Caen Caen France; ^4^ Department of Paediatrics CHRSM Namur Belgium; ^5^ Pediatric Allergy Unit Children's Hospital, University Hospital of Nancy Nancy France; ^6^ UR 3450 DevAH Health Faculty Nancy France; ^7^ Department of Pneumology and Allergology Strasbourg University Hospital Strasbourg France; ^8^ Allergology Office Saint‐Maur‐des‐Fossés France; ^9^ CH Roubaix Department of Paediatrics, Children's Hospital Roubaix France; ^10^ Pôle enfant, Hôpital Jeanne de Flandre CHU Lille, Pediatric Pulmonology and Allergy Department Lille France; ^11^ Univ Lille ULR 2694: Metrics Lille France; ^12^ Allergopôle, Clinique Saint‐Luc Namur Belgium

**Keywords:** buckwheat, emerging food allergen, goat's and sheep's milk, mandatory labelling food, peas and lentil, pine nut

## Abstract

**Background:**

The European Regulation list on mandatory labelling of foods includes 14 allergenic foods; however, other foods are also frequently implicated in food‐induced anaphylaxis (FIA).

**Methods:**

We analysed FIA cases reported to the Allergy Vigilance Network from 2002 to 2023. Allergenic foods involved in ≥ 1% of cases and not included in the list were assessed as emerging food allergens (EFA). We assessed their frequency, severity (Ring classification), recurrence, and potential presence in hidden form to determine which allergens might warrant inclusion on the list.

**Results:**

Among 2999 FIA cases (Ring grades 2–4), 413 cases (13.8%) met the selection criteria, divided into eight allergenic foods or food groups: goat's and sheep's milk (GSM, *n* = 84; 2.8% of the cases), buckwheat (*n* = 71; 2.4%), peas and lentil (*n* = 55; 1.8%), alpha‐gal (*n* = 50; 1.7%), pine nut (*n* = 49; 1.6%), kiwi (*n* = 44; 1.5%), beehive products (*n* = 30; 1.0%), and apple (*n* = 30; 1.0%). Severe reactions (Ring grades 3 and 4) were reported with GSM (46.8% and 4.8%, respectively, including two fatalities), buckwheat (46.5% and1.4%), peas‐lentil (20% and 1.8%), alpha‐gal (54% and 8%), and Grade 3 reactions were reported with pine nut in 49%, kiwi 54.5%, beehive products 33.3% and apple 46.7%. Recurrent reactions and hidden exposures were reported with GSM (56% and 15.5%), buckwheat (49.3% and 16.9%), peas‐lentil (7.3% and 9.0%) and pine nut (12.2% and 4.1%).

**Conclusion:**

We identified eight foods frequently involved in FIA and not currently listed in the European regulation. Given their frequency, severity, recurrence, and potential for hidden exposure, we propose that four—goat's and sheep's milk, buckwheat, peas‐lentil, and pine nut—be considered for inclusion in the list.

## Graphical abstract

Among eight allergenic foods without mandatory labelling involved in ≥ 1% of AVN database food anaphylaxis cases, four (goat's/sheep's milk, buckwheat, peas/lentils, pine nuts) should be considered for inclusion in the European mandatory labelling of foods list, due to their severity, recurrence, and sometimes hidden consumption.

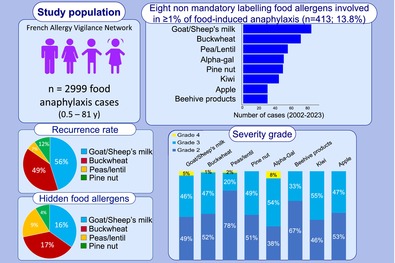

**Abbreviations**
Alpha‐gal: galactose‐alpha1,3‐galactose
AVN: Allergy‐Vigilance Network
CMA: cow's milk allergy
EFA: emerging food allergen(s)
FIA: food induced anaphylaxis
GSM: goat's milk and/or sheep's milk
MLF: mandatory labelling of foods
UPF: ultra‐processed food(s)

**Summary**

- Eight allergenic foods without mandatory labelling were each involved in ≥ 1% of food anaphylaxis cases.
- Four (goat's/sheep's milk, buckwheat, peas/lentil, pine nut) were associated with severe anaphylaxis.
- We propose that these four allergens be considered for inclusion in the European MLF list.

## 1 Introduction

In many countries, as the United States, Australia and the United Kingdom, the incidence of food‐induced anaphylaxis (FIA) increased in recent years with a lifetime prevalence estimated to be between 1.6% and 5.1% in the United States [1]. In children FIA, peanut is the key food involved in most countries, as in European countries. Besides peanut, data from the European Anaphylaxis Registry reported that cow's milk, cashew, hen's egg and hazelnut were also major food allergens triggering FIA in children. In adults, wheat flour, shellfish, hazelnut and soy are the most frequent elicitors of FIA [2]. The most frequent food allergens reported by the European Anaphylaxis registry all belong to the mandatory labelling of foods (MLF) list of 14 allergenic foods provided for by the European Regulation (EU) N.1169/2011 on the provision of food information to consumers (See Table S1). This list, established solely on the basis of the frequency of allergic reactions, has not been updated since 2011 [3]. The text provided for the possibility of a revision “in the case of the emergence of a risk to consumers' Health” (article 21), and indeed, several allergenic foods not included in the MLF list have been reported as elicitors of FIA by the European Anaphylaxis registry: other mammalian milks than cow's milk, other cereals than wheat, other legumes than peanut or soy, other seeds than sesame, fruits and vegetables other than celery [2].

The aim of our study was to determine the main allergenic foods triggering FIA besides those yet included in the MLF list, describe their characteristics, and alert the allergy community to the need to update this list, at least for some of them.

## 2 Methods

Since 2002, the Allergy‐Vigilance Network (AVN) collects all cases of anaphylaxis sent by its members (from French‐speaking countries, mainly France, Belgium and Luxembourg), from Grade 2–4 according to the Ring classification [4], using a structured questionnaire (online declaration form). This form contains over 70 items, including patient demographic and clinical data (age, gender, allergy history, allergenic food, date and place of occurrence), description of the anaphylaxis (clinical manifestations, severity of reaction), cofactors, epinephrine use, as well as the allergy assessment carried out (skin tests, specific IgE, etc.). All cases are validated by a panel of AVN allergy experts before being included in the database.

In this study, we focused on allergenic foods responsible for at least 1% of anaphylaxis cases reported to the AVN from 2002 to 2023, and not included in the MLF list. These are so‐called “emerging food allergens” (EFA) according to the French National Agency for Health Security ANSES (Agence Nationale de Sécurité Sanitaire) [5].

In addition to their frequency, we studied the severity following the Ring classification, the recurrence rate of the reaction, defined as the occurrence of symptoms related to the same culprit food before the declaration of anaphylaxis (allergy already known before declaration), and the possible hidden nature of the allergen, defined as a wrong or missing food labelling, an unexpected presence of the allergen in the food, or a contamination of the food by the allergen.

We also studied the evolution of the number of anaphylaxis cases for each allergenic food over four periods (2002–2007, 2008–2013, 2014–2018, 2019–2023).

The data has been anonymised in accordance with the current legislation of the French Commission for Data Protection. Consent was obtained from all participants or their relatives after an oral information session. This study was approved by the National Committee for Data and Patients Protection.

## 3 Statistics

Statistical analyses were performed with Graphpad (GraphPad Prism software version 8.0.0, San Diego, California USA). Chi‐square tests were used to perform comparisons of categorical data. The variation of each allergen of interest over the four time periods compared to the total FIA cases during these periods was studied using the Chi‐square test for trend.

## 4 Results

**FIGURE 1:**
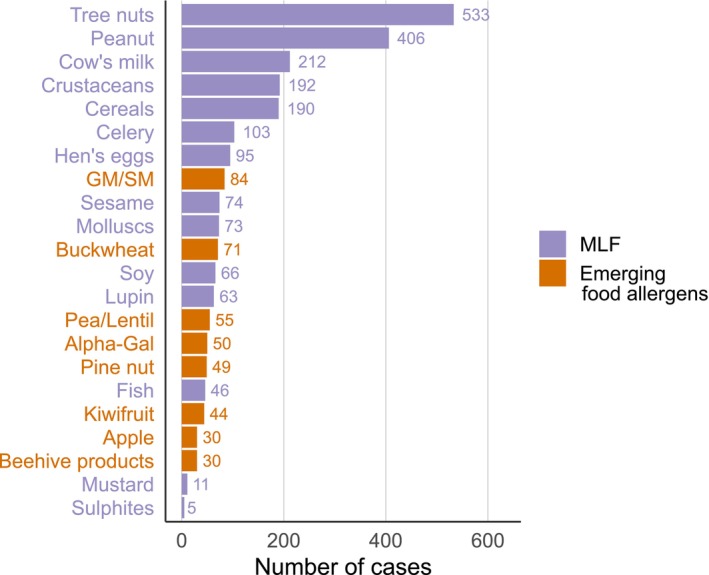
Distribution in decreasing order of frequency of the 14 foods with mandatory labelling and the eight emerging food allergens reported to the Allergy‐Vigilance Network (2002–2023).

**FIGURE 2:**
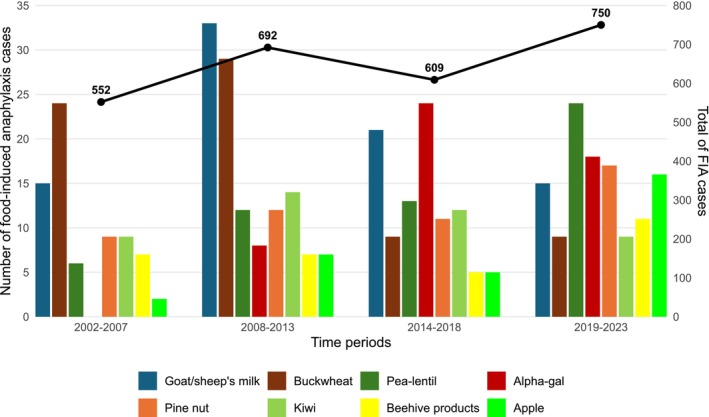
Number of anaphylaxis cases due to the eight emerging food allergens and total number of food‐induced anaphylaxis cases reported to the Allergy‐Vigilance Network, by time periods (2002–2023).

Of the 2999 FIA cases recorded by the AVN from 2002 to 2023, 1861 (62.1%) involved one of the 14 allergenic foods included on the European list and 413 (13.8%) involved EFA: goat's and/or sheep's milk (GSM, *n* = 84; 2.8%), buckwheat (*n* = 71; 2.4%), peas and/or lentil (*n* = 55; 1.8%), mammalian meat via galactose‐alpha1,3‐galactose (alpha‐gal) (*n* = 50; 1.7%), pine nut (*n* = 49; 1.6%), kiwi (*n* = 44; 1.5%), beehive products (*n* = 30; 1.0%) and apple (*n* = 30; 1.0%). Two allergens belonging to the European list were involved in less than 1% of the total FIA cases: mustard (*n* = 11; 0.4%) and sulphites (*n* = 5; 0.2%) (Figure 1). The main characteristics of EFA are detailed in Tables 1 and 2; their distribution by periods from 2002 to 2023 is presented in Figure 2.

**TABLE 1:** Anaphylaxis to emerging food allergens: Patients characteristics.

	Goat's‐sheep's milk	Buckwheat	Peas‐lentil	Alpha‐gal	Pine nut	Kiwi	Beehive products	Apple
Number of cases	84	71	55	50	49	44	30	30
% of total FIA cases	2.8	2.4	1.8	1.7	1.6	1.5	1.0	1.0
Sex‐ratio (M/F)	2.7	0.8	1.7	2.3	1.7	0.5	0.9	1.1
Median age, years (range)	10.0	30.0	3.0	50	12.9	8.0	35.0	25.0
	(0.6–66)	(2–77)	(0.6–79)	(10–81)	(1–73)	(2–72)	(10–64)	(4–61)
Children (< 18 year), *n* (%)	65 (77.4)	25 (35.2)	47 (85.5)	3 (6.0)	36 (73.5)	27 (61.4)	5 (16.7)	12 (40.0)
Atopy (%)	76.2	70.4	69.1	31.0	61.2	63.6	86.0	100
History of asthma, *n* (%)	35 (41.7)	26 (36.6)	8 (14.5)	4 (8.0)	17 (34.7)	11 (25.0)	6 (20.0)	9 (30.0)

Abbreviation: FIA, food‐induced anaphylaxis.

**TABLE 2:** Anaphylaxis to emerging food allergens: Clinical features.

	Goat's‐sheep's milk	Buckwheat	Peas‐lentil	Alpha‐gal	Pine nut	Kiwi	Beehive products	Apple
Total: *n* = 413	*n* = 84	*n* = 71	*n* = 55	*n* = 50	*n* = 49	*n* = 44	*n* = 30	*n* = 30
Severity grade 3 *n* (%)	39 (46.4)	33 (46.5)	11 (20.0)	27 (54.0)	24 (49.0)	24 (54.5)	10 (33.3)	14 (46.7)
Severity grade 4 *n* (%)	4 (4.8)*	1 (1.4)	1 (1.8)	4 (8.0)	0	0	0	0
Recurrence rate *n* (%)	47 (56.0%)	35 (49.3%)	4 (7.3)	33 (66.0)	6 (12.2)	12 (27.3)	5 (16.7)	6 (20)
Hidden allergen *n* (%)	13 (15.5)	12 (16.9)	5 (9.0)	0	2 (4.1)	0	0	0
Respiratory involvement, *n* (%)	64 (76.2)	47 (66.2)	35 (63.6)	24 (48.0)	33 (67.3)	27 (61.4)	19 (63.3)	18 (60.0)
Cardiovascular involvement, *n* (%)	37 (44.0)	32 (45.1)	6 (10.9)	33 (66.0)	13 (26.5)	10 (22.7)	10 (33.3)	10 (33.3)
Gastrointestinal involvement, *n* (%)	40 (47.6)	22 (31.0)	30 (54.5)	20 (40.0)	26 (53.1)	21 (47.7)	11 (36.7)	13 (43.3)
Epinephrine injection, *n* (%)	25 (29.8)	15 (21.1)	15 (27.3)	11 (22.0)	17 (34.7)	9 (20.5)	5 (16.7)	9 (30)
Cofactors, *n* (%)	12 (14.3)	19 (26.8)	4 (7.3)	25 (50.0)	12 (24.5)	7 (15.9)	11 (36.7)	18 (60)

* Including two deaths.

### 4.1 Goat's and/or Sheep's Milk

Most of the 84 GSM‐induced anaphylaxis cases were male (sex ratio: 2.7) and children (77.4%). Anaphylaxis was inaugural in 37 cases and 47 cases (56%) occurred in individuals with a history of GSM allergy (recurrent anaphylaxis). Only 14 patients (16.6%) had a history of cow's milk allergy (CMA), cured or still persistent. GSM‐induced anaphylaxis was severe, with a grade 3 reaction in 39 cases (46.4%) and a grade 4 reaction in 4 cases (4.8%), including two deaths at school. In 13 cases (15.5%), GSM was found in hidden form or as a contaminant: goat's cheese ravioli (*n* = 5), pizza (*n* = 5), cheese or milk (parmesan + UHT cow's milk), restaurant's cutlery and “flammeküche” (each 1 case). The rate of GSM‐induced anaphylaxis was stable during the study period, reaching 2% in 2019–2023 (Figure 2).

### 4.2 Buckwheat

Two‐thirds of the 71 cases involved adults. Occupational exposure to buckwheat was mentioned in 11 cases (15.5%): Six bakers, three crepe makers, two warehouse workers; one case occurred in a 15‐year‐old girl working as a bakery student. Buckwheat allergy was already known in 35 subjects (recurrence 49.3%), and anaphylaxis was severe, with 33 Grade 3 reactions (46.5%) and 1 grade 4 reaction (1.4%). Cofactors were reported in 19 cases (26.8%), mainly alcohol (7 cases) and exercise (5 cases). Buckwheat was consumed mainly in pancakes (*n* = 60; 84.5%) but was often a hidden, unexpected or contaminating allergen (*n* = 12; 16.9%). In two cases, anaphylaxis occurred following contact by inhalation.

There was a decrease in the rate of buckwheat‐induced anaphylaxis over time, ranging from around 4.3% of all FIA in the first two periods to around 1.2% for the last two periods (*p* < 0.001).

### 4.3 Peas and Lentil

Peas and lentil were analysed together because of their frequent cross‐reactivity, but even taken isolated, they nearly reached the level of 1%: of the 55 cases, 28 (50.9%) were due to peas (green peas 16 cases, blond peas 5, chickpeas 7) and 27 (49.1%) to lentil. Most cases occurred in children. A co‐sensitisation to peanut was mentioned in only 13 cases (23.6%), and to at least one other legume (pea, lentil, lupin, soy, chickpea) in 16 cases (29.1%). Anaphylaxis was inaugural in 25/28 pea cases and in 26/27 lentil cases and was moderately severe with 11 Grade 3 reactions (20.0%) and 1 Grade 4 (1.8%). Peas were consumed either in their natural form (*n* = 14; 50%) or as part of ultra‐processed food products (UPF; *n* = 14; 50%), while lentil was consumed in its natural form. The allergy was already known in four cases (7.3%) and hidden presence was mentioned in five cases (9.0%). The rate of peas‐lentil‐induced anaphylaxis increased during the study period from 1.1% of all FIA in the first period to 3.2% in the last period (*p* = 0.007).

### 4.4 Galactose‐alpha1,3‐Galactose (Alpha‐Gal)

Of the 50 cases of alpha‐gal‐induced anaphylaxis, 45 (90%) occurred in adults. Anaphylaxis was severe with 27 Grade 3 reactions (54%) and 4 grade 4 (8%), with cardiovascular involvement in 33 cases (66%). Cofactors were reported in 25 cases (50%), mainly concomitant medication (*n* = 12; 24%, including angiotensin‐converting enzyme inhibitors, sartans, beta‐blockers, gliptins, non‐steroidal anti‐inflammatory drugs, proton pump inhibitors), exercise (*n* = 6, 12%) and alcohol (*n* = 15; 30%). There were four occupational cases (8%): forestry occupations, landscaping activities and a slaughterhouse employee. A history of tick bites, past or recent, was reported in 25/50 cases. Foods triggering anaphylaxis were pork (*n* = 20, 40%; kidneys, andouillettes, sausages, pâté, food gelatin), and isolated consumption of mammalian meat in 20 cases (40%). One 74‐year‐old man experienced a Grade 3 reaction following a Geloplasma infusion. Allergy was already known in 33 cases (66%). No hidden consumption was observed. The first French case of alpha‐gal‐induced anaphylaxis was reported to the AVN in 2010, and the rate increased during the study period (2.4% of all FIA for the last period; *p* = 0.001).

### 4.5 Pine Nut

Of the 49 pine nut‐induced anaphylaxis, 36 (73.5%) occurred in children. There were 24 grade 3 reactions (49.0%). Anaphylaxis was inaugural in 43/49 cases (87.8%), and recurrent in 6 (12.2%). A concomitant allergy to peanut or tree nuts was reported in 8 individuals (16.3%). Cofactors were identified in 12 patients: exercise (*n* = 6), drugs (*n* = 3), alcohol (*n* = 2), and “stress” (*n* = 1). The food involved was pine nut in its natural form (25 cases), pesto sauce (11 cases), pastries (6 cases) and UPF (7 cases). The allergen was hidden in 2 cases (4.1%).

### 4.6 Kiwi

Of the 44 anaphylaxis cases induced by kiwi, 30 (68.2%) occurred in children. Anaphylaxis was inaugural in 32 cases (72.7%) and allergy was already known in 12 cases (27.3%). A co‐allergy was reported with birch pollen (*n* = 6; 13.6%) and grass pollen (*n* = 3; 6.8%). Green kiwifruit was the main culprit (*n* = 42; 95.5%; two cases with gold kiwi) with one case after a drop of fresh juice in the eyes. Reactions were rather severe, with 24 grades 3 (54.5%) but no grade 4.

### 4.7 Beehive Products

Of the 30 anaphylaxis cases induced by beehive products, 25 (83.3%) occurred in adults (median age 35 years; sex ratio 0.9). Only 5 subjects (16.7%) had a known allergy to the offending product before the anaphylaxis. Beehive products were pollen balls (*n* = 15; 50%), honey (*n* = 12; 40%), royal jelly (*n* = 2; 6.7%) and propolis (*n* = 1; 3.3%). As no specific IgE is available for these allergens, all cases have been confirmed by positive skin tests with the offending food in its native form. An allergic sensitisation to pollen was found in 26 subjects (86.7%) of whom 16 (53.3%) had seasonal rhinitis. All subjects with pollen balls‐induced anaphylaxis were sensitised to pollens: 13 to grass pollens (85.5%), 11 to Asteraceae pollens (73.3%), 5 to Betulaceae pollens (33.3%), whereas 9/12 cases of honey‐induced anaphylaxis (75%) were sensitised to pollens: 5 to grass pollens (41.6%), 8 to Asteraceae pollens (66.6%), and 3 to Betulaceae pollens (25%). Allergy was already known in 5 (16.7%), no hidden allergens were observed, and the severity was moderate with 20 grades 2 and 10 grades 3.

### 4.8 Apple

Of the 30 apple‐induced anaphylaxis cases, 18 (60%) occurred in adults. Anaphylaxis occurred frequently during the pollen season (*n* = 20; 66.6%); an allergic sensitisation to birch pollen was reported in 14 subjects (46.7%), cypress pollen in 6 (20%), and grass pollen in 2 (6.7%). A cofactor was reported in 18 cases: exercise (*n* = 15; 50%), concomitant medication, alcohol, and bypass surgery (each 1 case; 3.3%). Most reactions were induced by the consumption of raw apple (*n* = 28; 93.3%). PR‐10 sensitisation was found in 20 cases (66.6%), lipid transfer proteins in 7 (23.3%) and gibberellin‐regulated proteins in 3 (10.0%). Allergy to apple was already known in 6 (30%); no hidden consumption was reported, and the severity was moderate with 16 grades 2 and 14 grades 3. The rate of apple‐induced anaphylaxis increased significantly during the study period from 0.4% in the first period to 2.1% in the last period (16 cases; 53.3%; *p* = 0.006).

## 5 Discussion

In this study, eight foods or food groups not included in the MLF list were responsible for 1% or more of all FIA recorded by the AVN from 2002 to 2023. We called them emerging food allergens (EFA). Their frequency is higher than that of the two least frequent MLFs, mustard and sulphites.

Our results corroborate those of other studies. Baseggio Conrado et al. performed a systematic review and analysed 65 publications to provide data on the prevalence of FIA in 41 different countries [6]. Significant regional variations in the most common elicitors of FIA were evidenced. In this review, beehive products were not identified as FIA elicitors; GSM was included as “other mammalian milks” and alpha‐gal as “animal products” so that we cannot compare these data with our study. For the five remaining EFA identified by the AVN, the rate of pine nut anaphylaxis ranged from 0.5% in Canada to 3.9% in Korea, buckwheat from 0.2% (Canada) to 14% (Korea), legumes (excluding peanut, soy and lupin) from 0.6% (Portugal) to 4.4% (Italy), apple from 0.2% (Canada, Japan) to 5.3% (Italy), and kiwi from 0.6% (Israël) to 4.4% (Portugal). In Europe, according to the data from the NORA on 3427 FIA cases collected from 2007 to 2020 (including 948 cases transmitted by the French AVN), the rate of anaphylaxis induced by GSM was 1.3%, buckwheat 0.9%, peas‐lentil 1.3%, and pine nut, kiwi and apple 1.1% each [2]. No data were mentioned for alpha‐gal or beehive products. Thus, despite geographical disparities, the eight EFA identified by the AVN are frequently involved in FIA throughout the world and should not be considered as an “exception of French‐speaking countries”.

The relatively high frequency of anaphylaxis observed with some of our EFA can be explained by several elements, including dietary changes justified by supposedly healthier eating habits over the last decade (i. e. gluten‐free, dairy‐free, vegetarian, vegan), for environmental and animal welfare reasons, but also for health or even religious aspects. In these diets, consumption of a variety of plant foods is recommended to meet daily nutrients needs and prevent malnutrition [7]. Thus, the consumption of, for example, legumes (including peas and lentils), seeds (including pine nuts), buckwheat (in a gluten‐free diet), fruits, or beehive products increases, exposing consumers to new risks of allergy and anaphylaxis. Peas and lentils are increasingly consumed as meat substitutes and used by the food industry in UPF, due to their interesting emulsifying, solubilising, thickening properties [8, 9, 10]. They can be found as is, in a wide range of food products such as meat preparations, fish, sausages, sauces, ready‐to‐eat meals, pasta, chips, dairy products, ice creams, dietary supplements for athletes, or in processed form (i.e., pea fibres, pea isolates, pea flour, lentil flour) [10, 11, 12]. Sensitisation and allergy to legumes tend to be more frequent in regions where they are commonly consumed, such as European countries of the Mediterranean basin and Asian countries [12]. The potential risk of anaphylaxis induced by legumes, as highlighted for peas and lentil in our study, is also reinforced by the frequent co‐sensitisation or co‐allergy between legumes, especially with peanut with increasing number and severity of peanut‐induced anaphylaxis cases [13, 14]. However, co‐sensitisation to peanut was reported in only 23.6% of our peas‐lentil cases, reinforcing the fact that anaphylaxis from peas or lentils can occur independently of a known peanut allergy.

GSM anaphylaxis is often severe and unpredictable, with a high rate of recurrence. In the recently published paper of Pouessel et al., from our group, the specificities of GSM allergy have been studied in detail in 97 patients: GSM allergy begins later than CMA, it is rather rarely associated with CMA (13.4% in this large series, of which 3/4 were cured), GSM may present as an (unintentional) contamination of cow's milk, or in a masked form, and reactogenic doses are often very low [15]. These characteristics have been confirmed in other studies [16]. GSM is not a “French exception”, it has been described in Spain, Italy, Portugal, even in Canada [17], and it is possibly underreported.

Buckwheat allergy has also a high severity grade, a high rate of recurrence (49.3%) and hidden forms (16.9%). It is more often described in Asia than in Europe, where most of the work is case reports, but its prevalence in Europe might be underestimated; in an Italian work, 3.6% of 1954 patients were sensitised to buckwheat [18].

The role of alpha‐gal in anaphylaxis to red meat was established in 2009 and the involvement of tick bites was confirmed in 2011 [19]. In the United States, alpha‐gal anaphylaxis is currently a leading cause of food‐related anaphylaxis in adults [20]; this work highlights the main characteristics of this EFA, with delayed occurrence of anaphylaxis, frequency of co‐factors like exercise and alcohol, increase of alpha‐gal specific IgE after tick bites, with a six‐fold increase in alpha‐gal sensitisation between 2011 and 2018; cases may also occur with alpha‐gal containing drugs, or unexpected foods like desserts or puddings containing animal gelatines [21]. Consumption of alpha‐gal containing foods does not always trigger allergic reactions, and up to 80% of patients experience resolution of their symptoms with food avoidance. The high rate of recurrence in our series (66%) is explained by the fact that most people are not aware of a potential risk linked to red meat, and by the late onset of reactions. Once the diagnosis has been confirmed and explained, avoidance is relatively easy, and the allergen is rarely masked.

Pine nut anaphylaxis may also be severe: in a review of 45 cases of pine nut allergy, there were 34 cases of anaphylaxis (75%), one laryngeal angioedema and one asthma attack, and a high rate of monosensitisation [22]. In our series, two cases occurred after hidden consumption.

For kiwi, beehive products and apple, apart from their frequency and a few cases where the allergy was already known before the assessment, no other severity factor was highlighted (no grade 4, and no masked allergen in particular). These EFA were easily identified by patients and caregivers.

Combining the characteristics of the EFA anaphylaxis cases reported in our series—frequency and time trends, potential severity, recurrence rate and occurrence as hidden allergen—we believe that at least four of these eight EFA should be considered for inclusion in the European MLF list: GSM (2 deaths in our series), buckwheat, peas–lentil and pine nut. Their potential severity is also highlighted in other countries. Buckwheat caused severe and even lethal anaphylaxis in several countries [23, 24, 25, 26]. Legumes (excluding peanut and soybean) were responsible for 0.5 (adults) to 1.5% (children) of FIA fatalities in the UK [6]. Concerning pea anaphylaxis, an 8‐month‐old infant of our series had a grade 4 reaction with respiratory arrest confused with a convulsive attack. The recurrence rate, most often unmentioned in other studies, is high for these 4 EFA, ranging from 7.3% for peas–lentil to 56% for GSM. The same observation is made for the presence as a hidden allergen (16.9% for buckwheat and 15.5% for GSM in our series), underscoring the need for a better labelling legislation.

This need for better labelling does not seem as relevant for the other four EFA; because of its severity, the case of alpha‐gal could be discussed, but it is difficult to imagine how to include it in the MLF list; moreover, apart from a few exceptions, foods containing alpha‐gal are well known and easy to identify, which is also the case for kiwi, beehive products, and apple, consumed in their native form, without hidden presence in our series.

## 6 Limitations

The use of the old Ring classification in our series may seem surprising. However, since this work was undertaken in 2002, more modern and more suitable classifications such as that of the WAO or FASS were not available, and a retrospective reclassification of all these cases has not been carried out. In addition, the Ring classification is still the one used in the European NORA network, in which the AVN participates.

The AVN is an allergy monitoring tool; it is based on voluntary declarations from its members and therefore does not allow prevalence data to be established. Furthermore, it lists cases from French‐speaking European countries and may not be representative of other European countries.

## 7 Conclusion

This study highlights eight allergenic foods responsible for at least 1% of cases of food anaphylaxis, and not included in the European MLF list; given the severity and recurrence of anaphylaxis, and the possible presence of the allergen in masked form, we propose that four of them (goat's and sheep's milk, buckwheat, peas and lentils, and pine nuts) be considered for inclusion in the European mandatory labelling of foods list.

## Author Contributions

D.S.‐L., G.P. and X.B. analysed, interpreted the data and wrote the draft. S.T. collected data from the Allergy‐Vigilance Network. Y.K.A. performed the statistical analysis, interpreted the data, and reviewed the draft. D.S.‐L., D.M., E.B., A.D.‐C., E.B., C.M.F., P.B., P.D., J.S., Y.K.‐A., S.T., X.V.B, G.P. were major contributors in reviewing the draft and improving the paper with critical analysis. X.V.B. and G.P. were equal contributors for finalisation of work before submission. All authors read and approved the final manuscript.

## Conflicts of Interest

The authors declare no conflicts of interest.

## Supporting information


**Data S1:** cea70130‐sup‐0001‐Supinfo01.docx.

## Data Availability

The data that support the findings of this study are available on request from the corresponding author. The data are not publicly available due to privacy or ethical restrictions.
