# Supplementary material for: Food Anaphylaxis: Eight Food Allergens Without Mandatory Labelling Highlighted by the French Allergy‐Vigilance Network
Source: Clin Exp Allergy. 2025 Aug 20;55(11):1118–25. doi: 10.1111/cea.70130 (PMC12617491; doi:10.1111/cea.70130)
Supplement: Supplementary file 1 — Data S1: cea70130‐sup‐0001‐Supinfo01.docx. [file CEA-55-1118-s001.docx]

**Supplementary material**

Table S1. List of the 14 mandatory labelling allergens by alphabetic order^3^.

1. Cereals containing gluten, and products thereof;

2. Crustaceans and products thereof;

3. Eggs and products thereof;

4. Fish and products thereof;

5. Peanuts and products thereof;

6. Soybeans and products thereof;

7. Milk and products thereof (including lactose);

8. Nuts, namely: almonds (Amygdalus communis L.), hazelnuts (Corylus avellana), walnuts (Juglans regia), cashews (Anacardium occidentale), pecan nuts (Carya illinoinensis (Wangenh.) K. Koch), Brazil nuts (Bertholletia excelsa), pistachio nuts (Pistacia vera), macadamia or Queensland nuts (Macadamia ternifolia), and products thereof;

9. Celery and products thereof;

10. Mustard and products thereof;

11. Sesame seeds and products thereof;

12. Sulphur dioxide and sulphites at concentrations of more than 10 mg/kg or 10 mg/liter in terms of the total SO2;

13. Lupin and products thereof;

14. Molluscs and products thereof;
